# Genetic Dissection of Root Angle of *Brassica napus* in Response to Low Phosphorus

**DOI:** 10.3389/fpls.2021.697872

**Published:** 2021-07-29

**Authors:** Xianjie Duan, Xiaohua Wang, Kemo Jin, Wei Wang, Haijiang Liu, Ling Liu, Ying Zhang, John P. Hammond, Philip J. White, Guangda Ding, Fangsen Xu, Lei Shi

**Affiliations:** ^1^National Key Laboratory of Crop Genetic Improvement, Huazhong Agricultural University, Wuhan, China; ^2^Key Laboratory of Arable Land Conservation (Middle and Lower Reaches of Yangtze River), Microelement Research Centre, Ministry of Agriculture and Rural Affairs, Huazhong Agricultural University, Wuhan, China; ^3^College of Agriculture and Forestry Science, Linyi University, Linyi, China; ^4^Key Laboratory of Plant-Soil Interactions, College of Resources and Environmental Sciences, National Academy of Agriculture Green Development, Ministry of Education, China Agricultural University, Beijing, China; ^5^College of Resources and Environment, Hunan Agricultural University, Changsha, China; ^6^School of Agriculture, Policy and Development, University of Reading, Reading, United Kingdom; ^7^Southern Cross Plant Science, Southern Cross University, Lismore, NSW, Australia; ^8^The James Hutton Institute, Dundee, United Kingdom

**Keywords:** *Brassica napus*, lateral root angle, phosphorus, quantitative trait loci, genome-wide association study

## Abstract

Plant root angle determines the vertical and horizontal distribution of roots in the soil layer, which further influences the acquisition of phosphorus (P) in topsoil. Large genetic variability for the lateral root angle (root angle) was observed in a linkage mapping population (*Bna*TNDH population) and an association panel of *Brassica napus* whether at a low P (LP) or at an optimal P (OP). At LP, the average root angle of both populations became smaller. Nine quantitative trait loci (QTLs) at LP and three QTLs at OP for the root angle and five QTLs for the relative root angle (RRA) were identified by the linkage mapping analysis in the *Bna*TNDH population. Genome-wide association studies (GWASs) revealed 11 single-nucleotide polymorphisms (SNPs) significantly associated with the root angle at LP (LPRA). The interval of a QTL for LPRA on A06 (*qLPRA-A06c*) overlapped with the confidence region of the leading SNP (*Bn-A06-p14439400*) significantly associated with LPRA. In addition, a QTL cluster on chromosome C01 associated with the root angle and the primary root length (PRL) in the “pouch and wick” high-throughput phenotyping (HTP) system, the root P concentration in the agar system, and the seed yield in the field was identified in the *Bna*TNDH population at LP. A total of 87 genes on A06 and 192 genes on C01 were identified within the confidence interval, and 14 genes related to auxin asymmetric redistribution and root developmental process were predicted to be candidate genes. The identification and functional analyses of these genes affecting LPRA are of benefit to the cultivar selection with optimal root system architecture (RSA) under P deficiency in *Brassica napus*.

## Introduction

Oilseed rape (*Brassica napus* L., 2*n* = 38, genome AACC) is one of the most important oil crops for vegetable oil, feedstock, and biodiesel worldwide (Angelovič et al., 2013). Phosphorus (P) is an essential macronutrient for plant growth and development. P deficiency not only decreases the seed yield but also the oil production of *B. napus*. Modification of root system architecture in P-deficient soils is vital adaptation for plant P acquisition. Therefore, the breeding of P-efficient cultivars with an optimal RSA is an effective strategy for the genetic improvement of *B. napus* to reduce P fertilizer demand and maintain cultivar growth in soils with low P (LP) availability (Brown et al., 2013).

Under P deficiency, primary root elongation is strongly reduced while root hair and lateral root formation and growth are enhanced (Bates and Lynch, 1996; Lambers et al., 2006; Hammond et al., 2009; Niu et al., 2013). Agronomic P-use efficiency, physiological P-use efficiency, and P-utilization efficiency are correlated with root development and architecture traits, especially lateral root number (LRN) and length (LRL). In addition, P-efficiency ratio and physiology P-use efficiency had significant correlations with the lateral root angle, and agronomic P-use efficiency and P-utilization efficiency had significant correlations with growth rate at LP (Hammond et al., 2009). Previously, numerous quantitative trait loci (QTLs) and single nucleotide polymorphisms (SNPs) associated with RSA traits have been detected in plants grown under P deficiency (Wang et al., 2019). In common bean, at least three QTLs for basal root growth angle (root gravitropic traits) were associated with QTL for P-acquisition efficiency under LP availability in the field (Liao et al., 2004). In Arabidopsis, three QTLs involved in the root growth response to LP were mapped (Reymond et al., 2006). A total of seven QTLs were detected in maize for LRN and LRL at LP availability (Zhu et al., 2005). In an oilseed rape-recombinant inbred line (RIL) population, three QTL clusters, *uq.A1*, *uq.C3a*, and *uq.C3b*, associated with root traits (root length, root surface area, and root volume) were only observed under a suboptimal P supply (Yang et al., 2010). The *Bna*TNDH population, derived from a P-efficient cultivar, Ningyou7 and a P-inefficient cultivar, Tapidor, have previously been used to investigate the root morphological traits in both an agar-based growth system (Shi L. et al., 2013) and a “pouch and wick” high-throughput phenotyping (HTP) system (Zhang et al., 2016) under low phosphate (Pi) availability. A total of 14 QTLs associated with LRN, lateral root density (LRD), and primary root length (PRL) in the agar-based growth system was detected under LP availability. In the “pouch and wick” HTP system, a total of 34 QTLs associated with the total root length, the mean LRL, PRL, LRN, and LRD were detected under a suboptimal P supply. However, the phenotypic variability of the lateral root angle in response to P deficiency and the underlying molecular mechanisms in *B. napus* are unclear at present.

The angle of an organ maintained with respect to the gravity vector is known as the gravitropic set-point angle (GSA) (Digby and Firn, 1995). Most roots and shoots grow in non-vertical directions, and their growth angle is maintained through an antagonistic interaction between auxin-dependent gravitropic and antigravitropic offset components (Roychoudhry and Kepinski, 2015). The growth angle of lateral roots is also modified by the surrounding environment, including the cues related to water and nutrient availability, to grow vertically or radically into the ground (Niu et al., 2013). Under a low Pi medium, Arabidopsis show a more vertical orientation of lateral roots when plants grow on Petri plates (Bai et al., 2013; Roychoudhry et al., 2017). The basal roots of bean grow at a less vertical growth angle in low Pi levels, and the similar basal roots of the phenotype are also observed when plants grow on a low-auxin (50–70 nM) concentration medium while a higher concentration of auxin (90–100 nM) caused a more vertical orientation (Roychoudhry et al., 2017). Alterations in lateral root growth angle could be attributed to the modified auxin distribution in root tips (Singh et al., 2017). The branching and orientation of lateral roots directly influence the P absorption and acquisition by plants from soils with LP availability. The response of RSA to P deficiency is modified by plant hormones, where the changes in concentrations, sensitivity, and crosstalk mediate lateral root development, with plant hormones (such as auxin and ethylene) also playing an indispensable role in root gravitropism (Singh et al., 2017). A chemical genomics approach used to study the link between endomembrane system components and the gravitropic response in Arabidopsis, showed that 34 of 10,000 diverse chemicals could inhibit or enhance gravitropism, which were largely related to perception, signal transduction, and growth involved in the gravitropic response (Surpin et al., 2005).

To elucidate the genetic control of the lateral root angle (root angle) in response to P deficiency in *B. napus*, a linkage mapping population with 182 double haploid lines was used to detect the quantitative trait loci (QTLs) responsible for the root angle at an optimal P (OP) and a LP supply. In addition, an association panel containing 405 *B. napus* accessions was used to identify the significant SNPs associated with LPRA by genome-wide association studies (GWASs). The candidate genes within the CI of the QTL or QTL cluster were proposed.

## Materials and Methods

### Plant Materials

The *Bna*TNDH mapping population, including 182 doubled haploid (DH) lines, was used to detect QTLs for the root angle. The *Bna*TNDH population was developed by microspore culture from the F_1_ cross between women “Tapidor” (a European winter cultivar) and men “Ningyou7” (a Chinese semi-winter cultivar) (Qiu et al., 2006; Suwabe et al., 2008). Ningyou7 was characterized as a P-efficient cultivar with better growth and higher P acquisition than Tapidor in pot culture under normal P (200 mg P_2_O_5_/kg soil) and LP (20 mg P_2_O_5_/kg soil) supplies and in field trials under OP (90 kg P_2_O_5_/kg soil) and LP (9 kg P_2_O_5_/kg soil) conditions (Shi et al., 2010; Shi T. et al., 2013). In addition, a set of 405 diverse *B. napus*-inbred accessions, including 342 semi-winter, 34 spring, 26 winter, and three unknown types, were collected from the major breeding centers across China. Among them, 370 lines originated in China, 20 from Europe, five from Canada, four from Australia, four from Korea, and two from Japan (Wang et al., 2017).

### Plant Growth and Phenotypic Analysis

Root traits of 182 lines and the parent (Tapidor and Ningyou7) of the *Bna*TNDH population were screened by Zhang et al. (2016) in a “pouch and wick” HTP system with OP (0.25 mM Pi) and LP (0 mM Pi) conditions using quarter strength Hoagland’s solution. About 24 plants of each genotype were used to analyze the root growth angle of roots under OP and LP conditions. A diverse panel of 405 accessions was also screened in the “pouch and wick” HTP system previously at LP (0 mM Pi). For each accession, 16 seeds were separately sown across four different aluminum frames with four replicates in each tank and in different schedules (Wang et al., 2017). In this study, four sets of root phenotype data, such as the root angle in GWAS set 1 (GSRA1), root angle in GWAS set 2 (GSRA2), root angle in GWAS set 3 (GSRA3), and root angle in GWAS set 4 (GSRA4), were separately collected and analyzed. The RSA of all the plants in the two populations was imaged 14 days after sowing. The first-order lateral root angle (root angle) was manually calculated from these images using ImageJ (Schneider et al., 2012). Root angles were manually calculated by drawing a straight line through the root to obtain a mean trajectory of each lateral root (Guyomarc’h et al., 2012; Guseman et al., 2017). The root angle in this study is defined as the angular growth of lateral roots concerning the gravity vector (Digby and Firn, 1995), the vertical direction of roots is 0° (Supplementary Figure 1). In this study, the relative root angle (RRA) is calculated as the quotient of the root angle when plants are grown at a LP supply divided by the root angle when plants are grown at an OP supply.

### QTL Mapping

The development of molecular markers and the construction of genetic linkage groups were described by Zhang et al. (2016). The 19 linkage groups of “2041-map” with a length of 2,077.9 cM map distance and 1,698 SNP markers, 343 original markers, with a distance of 0.97 cM marker density on average. WinQTLCart v2.5 was used to detect significant QTLs for the root angle in OP and LP supplies and RRA and determine the additive effect of QTLs on the *Bna*TNDH population. The composite interval mapping (CIM) model was used to obtain the estimated additive QTLs and the percentage of phenotypic variation of each putative QTL. Walk speed was set as 1 cM. LOD (likelihood of odds) thresholds were calculated through the generation of 1,000 permutations. The LOD thresholds for the additive QTLs were set to 2.5 as the default manual input value. QTL-supported intervals were determined by two-LOD intervals around the QTL peak.

The QTL cluster was identified as two or more significant QTLs with overlapping CI. The QTLs for the root angle in this study and the reported traits in the “pouch and wick” HTP system, the agar system, and the field trials were integrated into a meta-analysis using the BioMercator v4.2 (Arcade et al., 2004). The Gerber and Goffinet’s (2000) meta-analysis model with the smallest Akaike information criterion (AIC) value was chosen for QTL integration. The principle of integration is that the peak position of component QTLs should be located within the CI of the integrated QTL.

### Genome-Wide Association Analysis

The association mapping panel of 405 *B. napus-*inbred lines is genotyped using the *Brassica* 60K Illumina^®^ Infinium SNP array, which contains 52,157 SNPs (Illumina Inc., San Diego, CA, United States). A total of 30,976 SNPs matched to a unique location in the reference genome of the cultivar Darmor-*bzh* and 19,397 high-quality SNPs with minor allele frequency (MAF) >0.05 and the call frequencies of SNPs < 0.8 (Wang et al., 2017) were selected to assess the population structure, relative kinship, and linkage disequilibrium (LD), and to conduct association analyses.

Marker-trait association analysis was performed by TASSEL 5.0 with the general linear model (GLM) procedures to control population structure (Q) and relative kinship (K). The general linear model (GLM) and the mixed linear model (MLM) were used to control population structure (Q) and relative kinship (K). Four mixed models: naïve model, Q model, K model, and Q + K model were applied to determine the statistical associations between phenotypes and genotypes. Quantile–quantile (QQ) plots were used for false-positive correction for association analyses. QQ plots and Manhattan plots were generated using the “CMplot” package in R. Significant associations between SNPs and traits were identified by scanning the genome with the value of *p* lower than the threshold *p* = 1/*N*, where *N* is the total number of SNP markers. The threshold of significance is set to *p* < 5.15 × 10^–5^.

### Detection of Candidate Genes

*Brassica napus* cultivar Darmor-*bzh* reference genome (Chalhoub et al., 2014) and the functional annotation of the Arabidopsis genome^1^ were used to identify candidate genes within the CIs. Based on the physical flanking marker positions of the QTLs, the genomic sequences of the QTL region were extracted. According to Wang et al. (2017), the LD decay in A subgenome is 250 kb and in the C subgenome is 1,100 kb on average. The candidate genes associated with the root angle were identified in the co-located interval region by both linkage mapping and GWAS analyses. Arabidopsis genes involved in root gravitropism were collected from previous studies (Supplementary Table 1). This study focuses on auxin-related genes and the genes involved in the root developmental process.

### Statistical Analyses

Restricted maximum likelihood (REML) procedures were used to estimate the source of variance, the missing values, and the correlations between traits using the GenStat version 19th. Heritability was also calculated as *H*^2^ = δ^2^g/(δ^2^g + δ^2^ ge/e + δ^2^/e × *r*), where δ^2^ g is the genetic variance, δ^2^ ge is genotype × environment interaction, δ^2^ is the error variance, *e* is the number of environments, and *r* is the number of replications per environment (Knapp et al., 1985; Sabadin et al., 2008) for an association panel.

## Results

### Significant Phenotypic Variation in the Lateral Root Angle Between the *Bna*TNDH Population and the Association Panel

In order to reduce the labor of lateral root angle measurement, we selected and measured the lateral root angles of 30 *B. napus* accessions from the 405 genotypes of the association panel. High positive correlations were observed among the average root angles of the first one, two, three, and four lateral roots on both the left and the right side of the primary root from hypocotyl (Table 1). The average root angle of the first four lateral roots on both the left and the right side of the primary root from hypocotyl had a significant positive correlation with the average root angle of all lateral roots (*r* = 0.94, Table 1 and Supplementary Table 2). Thus, it is feasible to measure the root angle of the first four lateral roots on both sides of the primary root from hypocotyl, to estimate the root angle of each genotype in the two populations.

**TABLE 1:** Correlations between the average lateral root angle of the first, second, third, fourth, and all first-order lateral roots on the left side and on the right side of the primary root in *Brassica napus* by a “pouch and wick” HTP system.

**Traits**	**ALLLR^a^**	**LR4**	**LR3**	**LR2**
LR4	0.94***			
LR3	0.90***	0.97***		
LR2	0.82***	0.91***	0.95***	
LR1	0.73***	0.81***	0.84***	0.90***

^*a*^ALLLR, average angle of all the first-order lateral roots; LR4, average angle of the first four first-order lateral roots on the left and right side; LR3, average angle of the first three first-order lateral roots on the left and right side; LR2, average angle of the first two first-order lateral roots on the left and right side; LR1, average angle of the first one first-order lateral roots on the left and right side of the primary root. Thirty cultivars or inbred lines are used in the correlation analysis. ***P ≤ 0.001.

**FIGURE 1:**
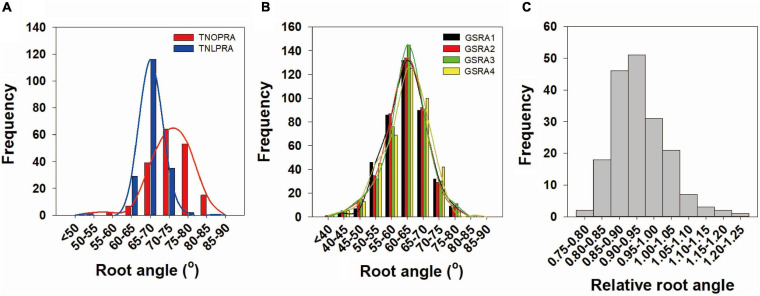
Frequency distribution of **(A,B)** the lateral root angle and **(C)** relative root angle in *Brassica napus*. **(A)** Lateral root angle of the *Bna*TNDH population at optimal phosphorus (OP) and low phosphorus (LP) supplies. **(B)** Lateral root angle of an association panel at LP supply. **(C)** Relative root angle in the *Bna*TNDH population. P, Phosphorus; TNOPRA, Root angle of *Bna*TNDH population at OP; TNLPRA, Root angle of *Bna*TNDH population at LP; GSRA1, Root angle in GWAS set 1; GSRA2, Root angle in GWAS set 2; GSRA3, Root angle in GWAS set 3; GSRA4, Root angle in GWAS set 4.

Extensive phenotypic variations in the root angle were observed in the *Bna*TNDH population at OP and LP supplies and in the association panel at a LP supply (Figure 1A and Table 2). Compared with the parent cultivar Ningyou7, the parent cultivar Tapidor had a significantly smaller root angle (deep root angle) (*p* = 0.018) at LP (Table 2). The root angle of *Bna*TNDH lines ranged from 59.6° to 83.6° at OP and from 60.1° to 76.9° at LP, and the average root angle of the *Bna*TNDH population was 72.8° at OP and 67.7° at LP (Table 2). The root angle of the accessions in the association panel of *B. napus* ranged from 40.8° to 77.0°. In addition, the coefficients of variation in different sets were constant, which ranged from 5.2 to 5.6% (Table 2). The root angle patterns in the *Bna*TNDH mapping population and the association panel of *B. napus* both fitted normal distributions. The broad-sense heritability of the association panel was 80.2% (Table 2). The spring-type cultivars (*n* = 34) had a significantly larger root angle (shallow root angle) compared with winter-type cultivars (*n* = 26) in the association panel (*p* = 0.034) (Figure 1 and Supplementary Table 3). A continuous phenotypic variance of RRA was observed in the *BnaTNDH* population (Figure 1C), and the coefficient of variation of RRA was 8.2%.

**TABLE 2:** Phenotypic variations for the lateral root angle in the *Bna*TNDH population and an association panel of *B. napus*.

**Population**	**P**	**Genotypes**	**Mean ± SD**	**Range**	**CV**	**H^2^**
	**levels**		**(°)**	**(°)**	**(%)**	**(%)**
*Bna*TNDH population	OP	Tapidor	61.9 ± 2.7	59.0–68.1	4.5	
		Ningyou7	64.2 ± 2.5	59.6–67.7	3.8	
		*Bna*TNDH population	72.8 ± 5.5	52.9–83.6	7.5	
	LP	Tapidor	59.0 ± 3.3	55.3–64.9	5.7	
		Ningyou7	62.5 ± 2.5	57.3–65.6	4.0	
		*Bna*TNDH population	67.7 ± 2.9	60.1–76.9	4.3	
An association panel of *B. napus*	LP	GSRA1	62.0 ± 6.5	40.7–79.8	5.2	80.2
		GSRA2	61.8 ± 6.6	40.0–79.7	5.4	
		GSRA3	62.2 ± 6.6	41.0–78.8	5.3	
		GSRA4	62.1 ± 7.0	39.0–80.4	5.6	

*OP, Optimal P; LP, Low P; GSRA1, Root angle in GWAS set 1; GSRA2, Root angle in GWAS set 2; GSRA3, Root angle in GWAS set 3; GSRA4, Root angle in GWAS set 4.*

### QTLs Associated With Lateral Root Angle and RRA

**FIGURE 2:**
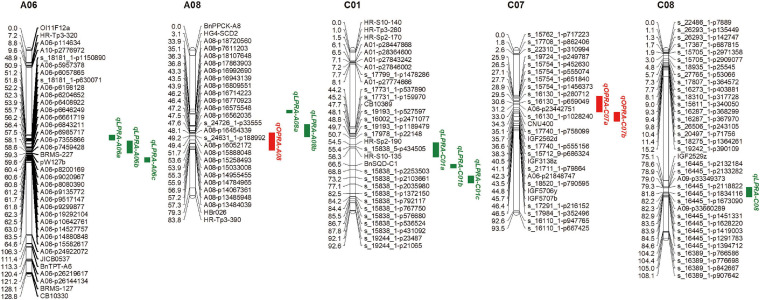
Quantitative trait loci (QTLs) associated with the lateral root angle in the “pouch and wick” HTP system of the *Bna*TNDH population at OP and LP, respectively. The QTL CIs are set as the map interval corresponding to a two-LOD decline on either side of the LOD peak. Red – QTLs associated with the lateral root angle at OP (OPRA) in the “pouch and wick” HTP system; Green – QTLs associated with the lateral root angle at LP (LPRA) in the “pouch and wick” HTP system.

Quantitative trait locus analysis was performed for the lateral root angle in the *Bna*TNDH population. Under OP, three QTLs associated with the lateral root angle were located on chromosomes A08 and C07 with *R*^2^ in the range of 5.4–7.9% (Figure 2). Among them, *qOPRA-A08* had a positive additive effect on the lateral root angle (Table 3), which indicated that the contribution of the QTL to the large lateral root angle phenotype was provided by Ningyou7. QTLs *qOPRA-C07a* and *qOPRA-C07b* had negative additive effects on the lateral root angle (Table 3), which indicated that the large lateral root angle phenotype was contributed by Tapidor.

**TABLE 3:** Quantitative trait loci (QTLs) associated with the lateral root angle of *B. napus* at OP and LP, respectively, by composite interval mapping (CIM).

**P treatment**	**QTL**	**Chromosome**	**Position (cM)**	**LOD score**	**CI (cM)**	**Additive effect**	***R*^2^ (%)**
OP	*qOPRA-A08*	A08	50.5	3.89	47.5–57.6	2.6287	7.9
	*qOPRA-C07a*	C07	28.9	3.46	26.5–35.7	−2.7569	6.9
	*qOPRA-C07b*	C07	39.3	2.53	35.7–41.0	−2.2452	5.4
LP	*qLPRA-A06a*	A06	50.9	3.53	48.9–51.8	2.4533	6.2
	*qLPRA-A06b*	A06	56.31	4.66	52.3–59.3	2.5146	8.1
	*qLPRA-A06c*	A06	62.41	3.67	61.8–64.5	3.4289	6.5
	*qLPRA-A08a*	A08	34.65	3.57	34.5–36.2	0.4473	6.2
	*qLPRA-A08b*	A08	44.65	3.84	42.8–49.9	0.4598	6.6
	*qLPRA-C01a*	C01	56.35	3.40	53.1–61.5	−1.4809	6.3
	*qLPRA-C01b*	C01	65.95	5.26	65.7–68.0	−1.5559	9.3
	*qLPRA-C01c*	C01	72.65	3.90	72.4–76.5	−1.4856	7.0
	*qLPRA-C08*	C08	84.01	2.80	79.0–84.5	−1.3991	4.8

*Each QTL was denominated as “q (QTL) + P treatment + RA (root angle) + chromosome + the serial letter.” A positive additive effect indicates a positive contribution of the Tapidor allele to the trait value, and a negative additive effect indicates a positive contribution of the Ningyou7 allele to the trait value.*

Under LP, nine QTLs associated with the lateral root angle were identified across the four chromosomes (A06, A08, C01, and C08) with *R*^2^ in the range of 4.8–9.3% (Figure 2). Three QTLs on chromosome A06 (*qLPRA-A06a*, *qLPRA-A06b*, and *qLPRA-A06c*) and two QTLs (*qLPRA-A08a* and *qLPRA-A08b*) on chromosome A08 all had positive additive effects with *R*^2^ > 6.0% (Table 3). Three QTLs on chromosome C01 and one QTL on chromosome C08 had negative additive effects (Table 3).

Relative root angle is used to evaluate the root angle plasticity of rapeseed in response to the LP condition. Five QTLs associated with RRA were identified by the QTL-mapping analysis in the *Bna*TNDH population, out of which four QTLs were located on chromosome A05 (*qRRA-A5a, qRRA-A5b, qRRA-A5c*, and *qRRA-A5d*) and one QTL (*qRRA-C07*) was located on chromosome C07. Among them, two QTLs *qRRA-A5a* and *qRRA-C07* had positive additive effects and three QTLs *qRRA-A5b, qRRA-A5c*, and *qRRA-A5d* had negative additive effects with *R*^2^ > 6.5% (Supplementary Table 4). Only one QTL *qRRA-C07* associated with RRA was co-located with QTL *qOPRA-C07a* associated with the root angle at OP (OPRA), indicating that QTL *qRRA-C07* only affect the OPRA.

In the current study, the QTL *qLPRA-C01a* (CI: 53.1–61.5 cM) associated with the lateral root angle under LP co-located with the QTL *RPC_LP_C01* (CI: 55.3–61.5 cM) associated with root P concentration under LP in agar culture (from Shi L. et al., 2013) and co-located with *qPRL_LP_REML_C1a* (CI: 44.6–54.5 cM) and *qPRL_LP_REML_C1b* (CI: 54.5–66.4 cM) associated with PRL under LP condition in the “pouch and wick” HTP system (Zhang et al., 2016). The QTL *qSY-LP1-C1b* (CI: 50.2–57.2 cM) associated with seed yield at LP in the field trial also co-located with the QTL *qLPRA-C01a*, *qPRL_LP_REML_C1a*, and *qPRL_LP_REML_C1b* (Shi T. et al., 2013; Zhang et al., 2016; Supplementary Figure 2). A QTL cluster located on chromosome C1 was identified by QTL meta-analysis, and its CI was estimated as 54.4–57.99 cM (Supplementary Table 5 and Supplementary Figure 2).

### Genome-Wide Association Analysis of Lateral LPRA

**FIGURE 3:**
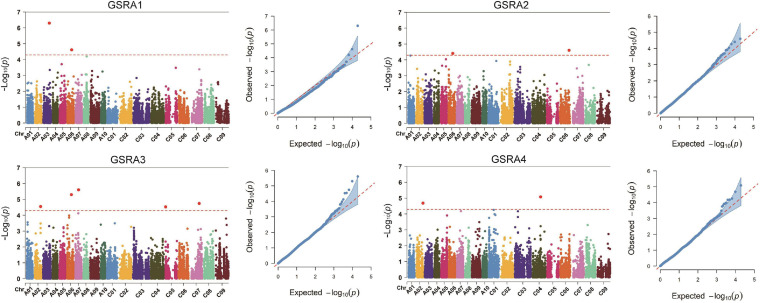
Quantile–quantile (QQ) and Manhattan plots for the root angle by genome-wide association study (GWAS). GSRA1, Root angle in GWAS set 1; GSRA2, Root angle in GWAS set 2; GSRA3, Root angle in GWAS set 3; GSRA4, Root angle in GWAS set 4.

Naïve model, Q model, K model, and Q + K model were used in association mapping across the four sets of experiments. According to the QQ plots of the four models, the observed −log_10_(*p*) values of the Q model were closer to the expected values of *p* than the other three models (Figure 3 and Supplementary Figure 3). A significant association between SNPs and traits is identified by scanning the genome with the threshold value of *p* < 5.15 × 10^–5^. A total of 11 SNPs located on eight of the 19 chromosomes were significantly associated with the lateral LPRA, and the phenotypic variation was explained by 7.90–10.68% (Table 4). Notably, the SNP of *Bn-A06-p14439400* was detected for the lateral root angles in GSRA1, GSRA2, and GSRA3 simultaneously, and explained the phenotypic variation by 8.6, 10.59, and 10.68%, respectively (Figure 3 and Table 4).

**TABLE 4:** Single-nucleotide polymorphisms (SNPs) associated with the lateral root angle of *B. napus* under LP by GWAS.

**Chromosome**	**Marker**	**Physical position (bp)**	**Alleles**	**MAF**	**Sets**	***P* value**	**−log_10_(*P*)**	**PVE (%)**
A02	*Bn-A02-p27179350*	24495096	A/C	0.0985	GSRA4	2.64E-05	4.58	8.86
A02	*Bn-A02-p23708117*	21917441	T/C	0.1404	GSRA3	2.85E-05	4.55	9.31
A03	*Bn-A03-p24016551*	22659038	T/G	0.1970	GSRA1	4.98E-07	6.30	9.10
A06	*Bn-A06-p14439400*	16012152	T/G	0.2833	GSRA1	2.43E-05	4.61	8.60
A06	*Bn-A06-p14439400*	16012152	T/G	0.2833	GSRA2	3.93E-05	4.41	10.59
A06	*Bn-A06-p14439400*	16012152	T/G	0.2833	GSRA3	5.03E-06	5.30	10.68
A07	*Bn-A07-p10873126*	12107078	T/G	0.2537	GSRA3	2.51E-06	5.60	9.67
C04	*Bn-scaff_15779_1-p181748*	30231788	T/G	0.0985	GSRA4	1.41E-05	4.85	7.96
C05	*Bn-scaff_16414_1-p1752434*	300846	T/C	0.1034	GSRA3	2.97E-05	4.53	9.23
C06	*Bn-scaff_17799_1-p2221144*	34328205	T/G	0.1650	GSRA2	2.52E-05	4.60	9.36
C07	*Bn-scaff_15705_1-p583113*	33980114	A/G	0.1355	GSRA3	1.81E-05	4.74	7.90

*GSRA1, Root angle in GWAS set 1; GSRA2, Root angle in GWAS set 2; GSRA3, Root angle in GWAS set 3; GSRA4, Root angle in GWAS set 4.*

### Prediction of Candidate Genes Affecting LPRA

The interval of QTL *qLPRA-A06c* on chromosome A06 overlapped with the interval of a peak SNP of *Bn-A06-p14439400* associated with the root angle in three of the four sets in the GWAS analysis (Figures 2, 3). The overlapping genomic region ranged from 62.35 to 63.54 Mb, which contained 87 genes (Supplementary Table 6). Eight of them were mainly associated with plant hormone signal transduction and response or related to the root developmental process, which were mapped to the *B. napus* reference genome of cultivar Darmor-*bzh* (Table 5). Among these genes, adenylate kinase 1 (ADK1), nucleoside diphosphate kinase 2 (NDPK2), flavonol synthase 3 (FLS3) and suppressors of PIN1 overexpression 1 (SUPO1), were associated with auxin signal regulation and response. One gene was associated with abscisic acid-activated signaling pathway, farnesylcysteine lyase (FCLY) and one gene, RING domain ligase 3 (RGLG3) related to jasmonic-mediated signaling pathway. The genes in the overlapped regions involved in the root development process were diacylglycerol kinase 2 (DGK2), RGLG3 (RING DOMAIN LIGASE 3), and phosphatidylinositol 4-OH kinase beta1 (PI-4KBETA1) (Table 5).

**TABLE 5:** Candidate genes for the lateral root angle of *B. napus* under LP by the linkage mapping and GWAS analysis.

**chr**	**Gene_id**	**Gene alias**	***A. thaliana* homologous gene**	**Gene start**	**Gene end**	**Root development**	**Gene annotation**	**Function description in Arabidopsis (TAIR)**	**References**
A06	GSBRNA2T00073134001	*BnaA06g22680D*	*AT5G63400*	15845949	15847970		Adenylate kinase 1 (*ADK1*)	Auxin mediated signaling pathway	Tan et al., 2011
A06	GSBRNA2T00073143001	*BnaA06g22750D*	*AT5G63590*	15948788	15950288		Flavonol synthase 3 (*FLS3*)	Flavonoid biosynthetic process	Turnbull et al., 2004
A06	GSBRNA2T00077276001	*BnaA06g23120D*	*AT5G63980*	16125732	16127813		SUPPRESSORS OF PIN1 OVEREXPRESSION 1 (*SUPO1*)	Auxin mediated signaling pathway; Abscisic acid-activated signaling pathway; regulation of jasmonic acid biosynthetic process	Zhang et al., 2011
A06	GSBRNA2T00073128001	*BnaA06g22660D*	*AT5G63310*	15819987	15821809		Nucleoside diphosphate kinase 2 (NDPK2)	Auxin-activated signaling pathway	Choi et al., 2005
A06	GSBRNA2T00077268001	*BnaA06g23050D*	*AT5G63910*	16089593	16091935		Farnesylcysteine lyase (FCLY)	Abscisic acid-activated signaling pathway	Huizinga et al., 2010
A06	GSBRNA2T00077275001	*BnaA06g23110D*	*AT5G63970*	16123073	16124988	Yes	RING DOMAIN LIGASE 3 (RGLG3)	Jasmonic acid mediated signaling pathway	Zhang et al., 2012
A06	GSBRNA2T00073162001	*BnaA06g22880D*	*AT5G63770*	16021030	16024591	Yes	Diacylglycerol kinase 2 (DGK2)	NAD+ kinase activity, diacylglycerol kinase activity, Response to root elongation and plant development	Gómez-Merino et al., 2005
A06	GSBRNA2T00077289001	*BnaA06g23220D*	*AT5G64070*	16163866	16170987	Yes	Phosphatidylinositol 4-OH kinase beta1 (*PI-4KBETA1*)	1-phosphatidylinositol 4-kinase activity	Rubilar-Hernández et al., 2019
C01	GSBRNA2T00048739001	*BnaC01g14670D*	*AT4G23130*	9943307	9944811	Yes	Cysteine-rich RLK (RECEPTOR-like protein kinase) 5 (CRK5)	Receptor-like protein kinase	Rigó et al., 2013
C01	GSBRNA2T00022466001	*BnaC01g15360D*	*AT4G23640*	10524195	10527820	Yes	Tiny root hair 1 (TRH1)	Potassium transporter	Vicente-Agullo et al., 2004
C01	GSBRNA2T00116184001	*BnaC01g15800D*	*AT4G23980*	10827454	10830328	Yes	Auxin response factor 9 (ARF9)	Auxin response factor	Liu et al., 2008
C01	GSBRNA2T00116104001	*BnaC01g16320D*	*AT4G24390*	11209843	11212502	Yes	Auxin Signaling F-BOX 4 (AFB4)	Auxin-activated signaling pathway	Greenham et al., 2011
C01	GSBRNA2T00116207001	*BnaC01g15610D*	*AT4G23750*	10709497	10710466	Yes	Cytokinin response factor 2 (CRF2)	A member of the ERF (ethylene response factor) subfamily	Waidmann et al., 2019
C01	GSBRNA2T00116231001	*BnaC01g15500D*	*AT5G03730*	10612895	10614282	Yes	Constitutive triple response 1 (CTR1)	Negative regulation of ethylene-activated signaling pathway	Negi et al., 2008; Qin and Huang, 2018
									

A total of 192 genes were identified within the CI (54.40–57.99 cM) of QTL cluster1, which were associated with the root angle, PRL, root P concentration, and seed yield on chromosome C01 in the *Bna*TNDH population (Supplementary Table 7). Six genes were predicted to be candidate genes. Among them, tiny root hair 1 (TRH1) and cysteine-rich RLK (RECEPTOR-like protein kinase) 5 (CRK5) have been reported to be in association with root gravitropism modulation (Vicente-Agullo et al., 2004; Rigó et al., 2013; Supplementary Table 1). Auxin response factor 9 (ARF9) and auxin signaling F-BOX 4 (AFB4) were associated with auxin signal transduction and response (Liu et al., 2008; Greenham et al., 2011). Cytokinin response factor 2 (CRF2) was associated with cytokinin signal response, and constitutive triple response 1 (CTR1) was related to the negative regulation of an ethylene-activated signaling pathway (Waidmann et al., 2019).

## Discussion

Increasing the distribution of lateral roots in the topsoil where P availability can be higher can benefit P acquisition by plants (White et al., 2013). Root angle is an important root trait, and uncovering the genetic mechanism of the root angle is essential for the breeding of P-efficient *B. napus* cultivars (Duan et al., 2020). In this study, the *Bna*TNDH population and an association panel of *B. napus* were used to identify the QTLs and SNPs associated with the lateral root angle under OP and LP conditions in the “pouch and wick” HTP system.

### Lateral Root Angle in Response to P Deficiency

It is difficult to observe and quantify the root growth angle in a non-destructive manner in the field. Thus, in this study, the “pouch and wick” HTP system was employed to study the root angle of *B. napus* at both OP and LP supplies. In contrast to other studies, the lateral root angle of the *Bna*TNDH population is reduced, that is, the lateral root became deeper under LP compared with OP (Figure 1). This could be attributed to the distribution of P within the growing system, with the nutrients fed from the bottom of the system and wicking up the filter paper, potentially resulting in greater P availability lower in the root zone profile (Bates and Lynch, 1996). A similar result was also reported in Arabidopsis seedlings, with more roots growing vertically in low Pi medium relative to normal Pi medium (Bai et al., 2013). However, in the field, the majority of common bean genotypes had a shallow LPRA compared with high P (Liao et al., 2004). Plant roots prefer to absorb P with a shallow root angle in the field because of more P concentrated in the topsoil and prefer to uptake more P with a steep root angle from the tank in the HTP system because of the nutrients fed from the bottom (Huang and Zhang, 2020). Internal and external P concentration acts as local and systemic signals to alter the root architecture system through participating in the signal transduction pathway to control root set-point angle (Pérez-Torres et al., 2008; Roychoudhry et al., 2017). In Arabidopsis, GSA of roots in P-deprived plants is mediated by auxin concentration in the root tips, influenced by TIR1 and AFB3 in transcript and protein levels, which through changing auxin transporter activity, rather than auxin synthesis, regulates lateral root GSA (Pérez-Torres et al., 2008). At LP, winter-type *B. napus* cultivars had significantly smaller root angles (deep roots) compared with spring-type cultivars in the association panel (Figure 1). Relatively shorter values of TRL, LRL, and PRL were also observed in winter-type cultivars compared with spring-type cultivars at LP (Wang et al., 2017).

### Genetic Loci Associated With Lateral Root Angle in Response to P Deficiency

The identification of QTLs for target traits is critical for the breeding of P-efficient crop varieties through a combination of linkage mapping and association mapping methods. In this study, three QTLs associated with the lateral OPRA were mapped to chromosomes A08 and C07, and nine QTLs associated with lateral LPRA were mapped to chromosomes A06, A08, C01, and C08 using the linkage mapping analysis (Table 3). In addition, a total of nine significant SNPs associated with lateral LPRA were located on *B. napus* chromosomes A02, A03, A06, A07, C04, C05, C06, and C07 using GWAS (Table 4). A recent GWAS of the lateral root angle of *B. napus* in the field using a shovelomics method identified a total of eight significant SNPs, which were located on chromosomes A02, A03, A09, C03, C06, and C09 (Arifuzzaman et al., 2019). There were no overlapping intervals between the QTLs for lateral OPRA or LP identified in the “pouch and wick” HTP system and the significant SNPs for the lateral root angle identified in the field as the genetic population, sampling period, and growth medium between the two studies were different. Moreover, there were also no overlapping intervals of the significant SNPs associated with the lateral root angle between the GWAS population in this study and in a study by Arifuzzaman et al. (2019) due to the difference of the cultivars, sampling period, growth medium, especially P distribution in the growth medium, between the two GWAS populations. In the field, the heterogeneous distribution of nutrients in the soil leads to roots proliferating in the areas of soil with high P concentrations, and the environmental variance can directly increase the additive genetic variance of QTLs (Gorelick, 2005). The nutrients were distributed in the lower root zone profile in the “pouch and wick” HTP system, and the roots absorbed the nutrients from the bottom.

### Candidate Genes Underlying the QTL *qLPRA-A06c* and the QTL Cluster1

Identifying the specific genes controlling P-efficiency-related traits underlying the genetic loci is critical for the breeding of future P-use efficient crops (Wang et al., 2019). In this study, 87 genes were located in the genomic region of the co-located CIs of the QTL *qLPRA-A06c* and the LD decay region of the significant SNP *Bn-A06-p1443940* associated with lateral LPRA on chromosome A06 (Supplementary Table 6). The biological functions of the orthologs of eight candidate genes in Arabidopsis were involved in root angle modification or root development processes. Four genes were involved in the auxin signaling pathway (Table 5). *BnaA06g22680D* encodes an adenylate kinase 1 (*ADK1*), and its protein levels were increased in Arabidopsis root tips when plants under horizontal treatment for 12h, however, the enzyme activity of ADK1 in pin2 mutant was insensitive. The changes of ADK1 may be associated with an auxin-mediated early phase gravity signaling event in root cap cells (Tan et al., 2011). *BnaA06g22750D* encodes a *FLS3* and involves in the flavonoid and flavonol biosynthesis, and under Pi deficiency, tobacco plants accumulate more flavonols (Turnbull et al., 2004; Jia et al., 2015), and nanomolar concentrations of flavonols application could partially restore the root gravitropism of *pin2* mutant plants mainly through redressing the formation of lateral auxin gradients (Santelia et al., 2008). *BnaA06g23120D* encodes *SUPO1*, which is a PIN-mediated auxin transport regulator. The mutant of *BnaA06g23120D* is defective in inositol phosphatase (*SAL1*) and has increased InsP_3_ and cytosolic Ca^2+^ levels, which further affect PIN polar targeting and auxin distribution (Zhang et al., 2011). *BnaA06g22660D* encodes a *NDPK2*, which participates in auxin-regulated processes through modulation auxin transport (Choi et al., 2005). Apart from the auxin-related gene, we also found one gene described as affecting abscisic acid (ABA) signaling transformation, *BnaA06g23050D*, which encodes a *FCLY*, and its mutant showed an ABA hypersensitive phenotype because of the accumulation of farnesylcysteine and the inhibition of isoprenylcysteine methyltransferase (Huizinga et al., 2010). *BnaA06g23110D* encodes a ubiquitin ligase containing a RING domain, and the altered expression of *RGLG3* and *RGLG4* affected methyl jasmonic acid- (JA-) inhibited root growth and JA-inductive gene expression (Zhang et al., 2012). Two genes are involved in lateral root development. Among them, *BnaA06g22880D* codes for a *DGK2* and inhibiting its activity seriously limit root elongation and lateral root growth (Gómez-Merino et al., 2005). *BnaA06g23220D* codes for a *PI-4KBETA1* promote lateral root organogenesis through the regulation of phosphatidylinositol 4-phosphate biosynthesis (Rubilar-Hernández et al., 2019).

In addition, 192 genes were identified in the CI of QTL cluster1 associated with the lateral root angle, PRL, root P concentration, and seed yield at LP on chromosome C01 (Supplementary Table 7). Among them, *BnaC01g14670D* encodes a *CRK5*, which is required for the proper polar localization of PIN2 in the transition zones of roots (Rigó et al., 2013). *BnaC01g15360D* encodes a *TRH1*, which plays an important role in auxin translocation in the root cap. The auxin transport through the root cap in *trh1* mutant is blocked, which reduces the overall acropetal auxin transport and decreases auxin concentration in the cortex/epidermis, and results in root gravitropic defect (Vicente-Agullo et al., 2004). *BnaC01g15800D* encodes an ARF9, and double knock out of ARF9 and ARF13 lines shows that two genes redundantly control suspensor development and the suspensor-specific IAA10/ARF13/ARF9 auxin response machinery is required for root formation (Liu et al., 2008). *BnaC01g16320D* encodes an AFB4, and its mutant has shorter roots and produced more lateral roots/PRL (Greenham et al., 2011). *BnaC01g15610D* encodes a CRF2, and the asymmetric increase of cytokinin signaling in the upper flank of lateral roots in stage II modulates root gravitropic bending (Waidmann et al., 2019). *BnaC01g15500D* encodes a CTR1, which is related to constitutive ethylene-signaling response. The mutant of *BnaC01g15500D* showed less emerged lateral root number and short PRL (Negi et al., 2008; Qin and Huang, 2018).

In this study, only 14 candidate genes involved in root development were selected for further study. However, other genes may also be associated with the root angle modification at LP. Bulk segregant analysis sequencing (BSA-seq) could be used to narrow down the target region of the major QTL and mine the genes that control the root angle under the LP condition of *B. napus*.

## Conclusion

A smaller lateral root angle was found in the *B. napus* at LP than at OP in the “pouch and wick” HTP system, indicating that the distribution of P in the growth medium decided RSA. A QTL on chromosome A06, *qLPRA-A06c*, associated with the lateral root angle was identified by the linkage mapping analysis and GWAS analysis simultaneously at LP. A QTL cluster on chromosome C01 at LP was associated with the lateral root angle, PRL, root P concentration, and seed yield concurrently. The candidate genes within the CIs were proposed. Illustration of QTLs and underlying genes in the controlling LPRA will be helpful for the dissection of the P-efficiency mechanism, which is further helpful for cultivar selection with an optimal RSA to facilitate P acquisition under P deficiency in *B. napus*.

## Data Availability Statement

The datasets presented in this study can be found in online repositories. The names of the repository/repositories and accession number(s) can be found in the article/Supplementary Material.

## Author Contributions

XD, XW, WW, and HL contributed to design of the study. XD, LL, and YZ contributed to data collection. XD and XW performed the statistical analysis. XD wrote the first draft of the manuscript. JH, PW, GD, FX, and LS contributed on writing – review and editing of the manuscript. All authors contributed to manuscript revision, read, and approved the submitted version.

## Conflict of Interest

The authors declare that the research was conducted in the absence of any commercial or financial relationships that could be construed as a potential conflict of interest.

## Publisher’s Note

All claims expressed in this article are solely those of the authors and do not necessarily represent those of their affiliated organizations, or those of the publisher, the editors and the reviewers. Any product that may be evaluated in this article, or claim that may be made by its manufacturer, is not guaranteed or endorsed by the publisher.

## Footnotes

1
https://www.arabidopsis.org

## Supplementary Material

Supplementary Figure 1 The measure method of the lateral root angle of *Brassica napus*. The horizontal direction is 90° and the vertical direction is 0°.

Supplementary Figure 2 Meta-analysis of a quantitative trait locus (QTL) cluster on chromosome C01 for the root angle, primary root length (PRL) in the “pouch and wick” HTP system, the root P concentration in the agar system, and the seed yield in field trials in the *Bna*TNDH population.

Supplementary Figure 3 The quantile–quantile (QQ) plot for the lateral root angle by a genome-wide association study (GWAS). GSRA1, Root angle in GWAS set 1; GSRA2, Root angle in GWAS set 2; GSRA3, Root angle in GWAS set 3; GSRA4, Root angle in GWAS set 4.

Supplementary Table 1 Key genes involved in root gravitropism in Arabidopsis.

Supplementary Table 2 Root angle of all the first-order lateral roots of 30 genotypes of *Brassica napus.*

Supplementary Table 3 Root angle of the first-order lateral roots in the *Bna*TNDH population at optimal phosphorus (OP) and low phosphorus (LP) and an association panel of *B. napus* at LP.

Supplementary Table 4 Quantitative trait loci (QTLs) associated with the relative root angle (RRA) of the *Bna*TNDH population in the “pouch and wick” HTP system, by composite interval mapping (CIM).

Supplementary Table 5 Meta-analysis of a cluster associated with the lateral root angle, primary root length (PRL), root P concentration, and seed yield identified in the *Bna*TNDH population.

Supplementary Table 6 The genes in the co-located region on chromosome A06 for the root angle of *B. napu*s were detected by the QTL mapping and GWAS analysis at LP.

Supplementary Table 7 The genes within the CI of cluster1 on chromosome C01 for the lateral root angle, PRL, root P concentration, and seed yield of *Bna*TNDH population at LP.
