# Supplementary figures and images for: Genetic Dissection of Root Angle of Brassica napus in Response to Low Phosphorus
Source: Front Plant Sci. 2021 Jul 29;12:697872. doi: 10.3389/fpls.2021.697872 (PMC8358456; doi:10.3389/fpls.2021.697872)

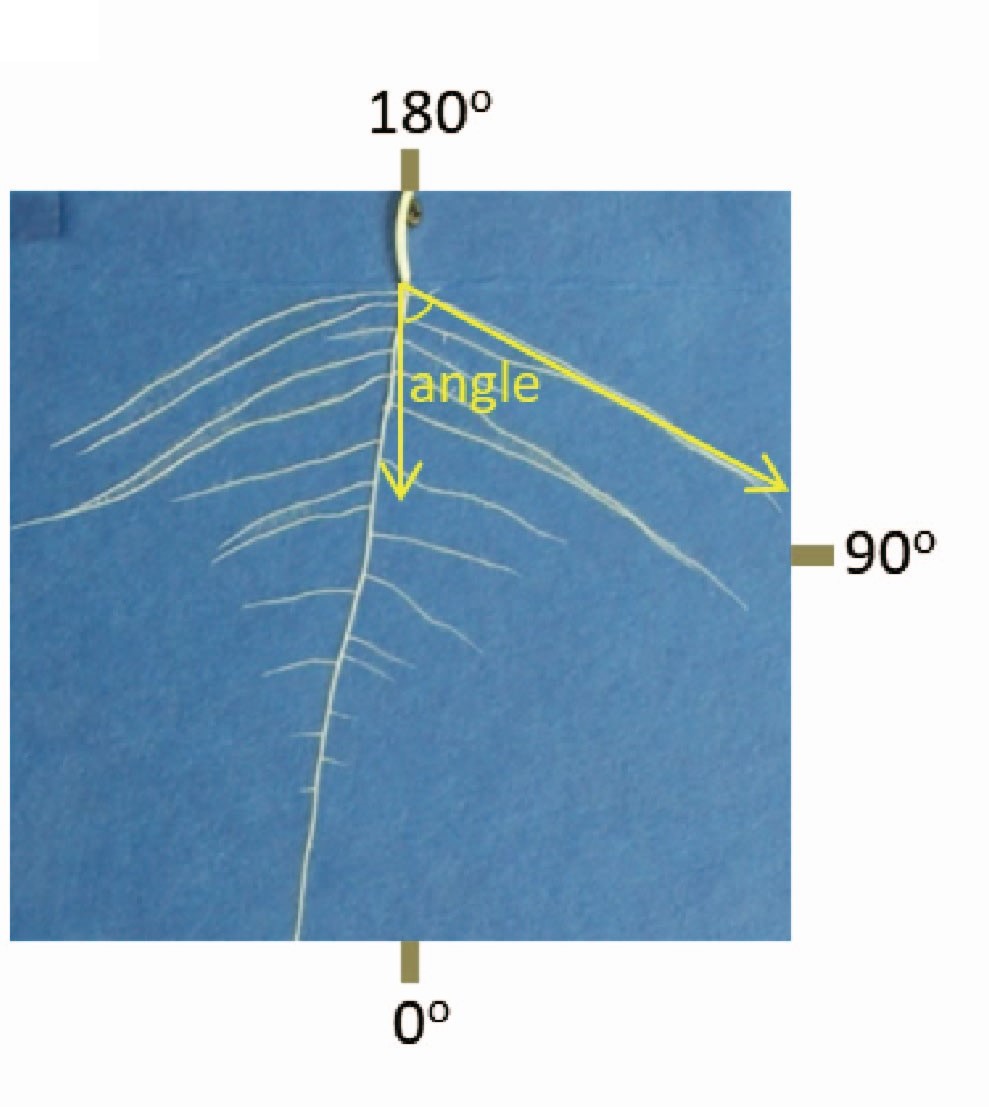

Supplement: Supplementary Figure 1 — The measure method of the lateral root angle of Brassica napus. The horizontal direction is 90° and the vertical direction is 0°. [file Image_1.JPEG]

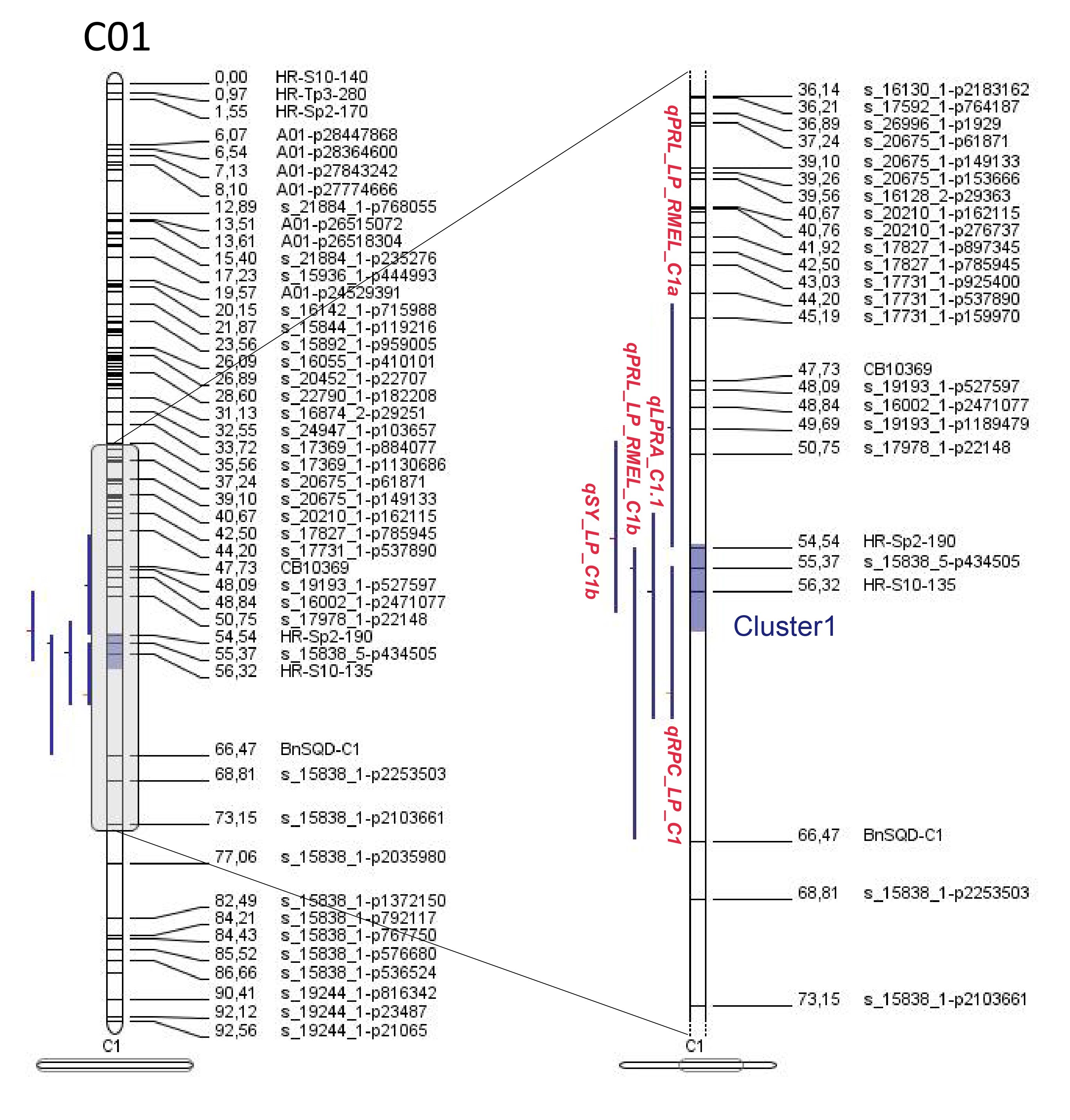

Supplement: Supplementary Figure 2 — Meta-analysis of a quantitative trait locus (QTL) cluster on chromosome C01 for the root angle, primary root length (PRL) in the “pouch and wick” HTP system, the root P concentration in the agar system, and the seed yield in field trials in the BnaTNDH population. [file Image_2.JPEG]

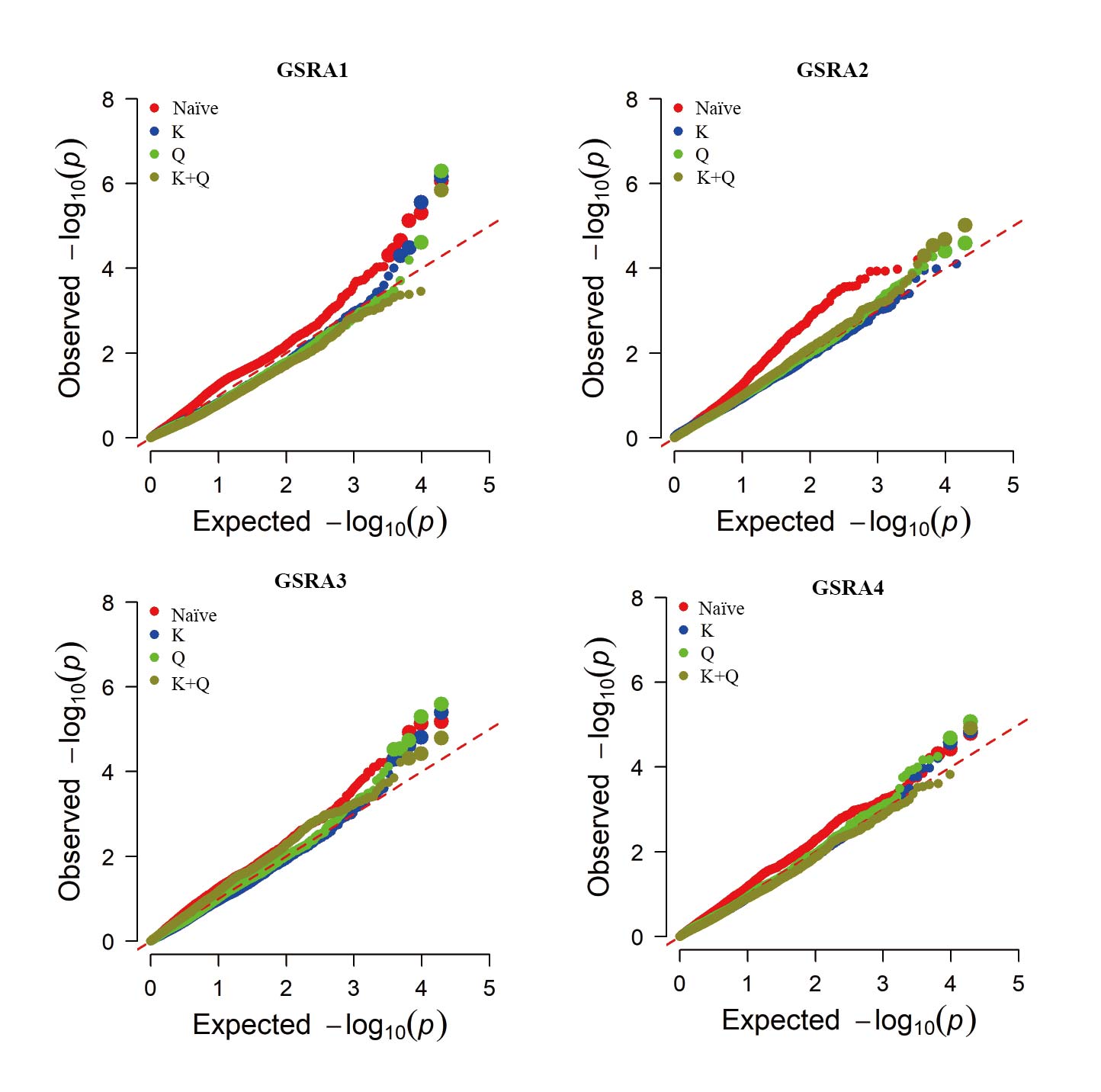

Supplement: Supplementary Figure 3 — The quantile–quantile (QQ) plot for the lateral root angle by a genome-wide association study (GWAS). GSRA1, Root angle in GWAS set 1; GSRA2, Root angle in GWAS set 2; GSRA3, Root angle in GWAS set 3; GSRA4, Root angle in GWAS set 4. [file Image_3.JPEG]
